# High Percentage of Complications and Re-Operations Following Dynamic Locking Plate Fixation with the Targon^®^ FN for Intracapsular Proximal Femoral Fractures: An Analysis of Risk Factors

**DOI:** 10.3390/medicina58121812

**Published:** 2022-12-09

**Authors:** Emanuel Kuner, Jens Gütler, Dimitri E. Delagrammaticas, Bryan J. M. van de Wall, Matthias Knobe, Frank J. P. Beeres, Reto Babst, Björn-Christian Link

**Affiliations:** 1 Department of Orthopedic and Trauma Surgery, Lucerne Cantonal Hospital, 6000 Lucerne, Switzerland; 2 Department of Orthopedics and Traumatology, Zug Cantonal Hospital, 6340 Baar, Switzerland; 3 Central Coast Orthopedic Medical Group, 862 Meinecke Avenue, Suite 100, San Luis Obispo, CA 93405, USA

**Keywords:** avascular, band, femoral, fracture, neck, necrosis, iliotibial, implant, osteosynthesis

## Abstract

The ideal surgical treatment of femoral neck fractures remains controversial. When treating these fractures with internal fixation, many fixation constructs exist. The primary aim of this study was to evaluate the incidence and specific risk factors associated with complication and re-operation following fixation of intracapsular proximal femoral fractures using the Targon-FN system (B.Braun Melsungen AG). A secondary aim was to identify if lateral prominence of the implant relative to the lateral border of the vastus ridge was a specific risk factor for elective plate removal. Methodically, a retrospective case series was conducted of all consecutive adult patients treated at a single level 1 trauma center in Switzerland for an intracapsular proximal femoral fracture with the Targon-FN. Demographic data were collected. Patients with a follow-up of less than three months were excluded. Complications as well as plate position were recorded. Statistical analysis to identify specific risk factors for re-operation and complications was performed. In result, a total of 72 cases with intracapsular femoral neck fractures were treated with the Targon-FN locking plate system between 2010 and 2017. Thirty-four patients (47.2%) experienced one or more complications. The most common complication was mechanical irritation of the iliotibial band (ITB) (23.6%, *n* = 17). Complications included intraarticular screw perforation (6.9%, n = 5), avascular necrosis (5.6%, n = 4), non-union (5.6%, n = 4) among others. In total, 46 re-operations were required. Younger age, fracture displacement and time to postoperative weight bearing were identified as risk factors for re-operation. In conclusion, intracapsular femoral neck fractures treated with the Targon-FN system resulted in a high rate of post-operative complication and re-operation. Statistical analysis revealed patient age, fracture displacement, time to postoperative full weight bearing were risk factors for re-operation. The main limitation is the limited number of cases and a short follow-up of less than 12 months in a subgroup of our patients.

## 1. Introduction

Femoral neck fractures are among the most common orthopaedic injuries treated in the elderly population [1]. In patients over 65 years of age, the incidence of femoral neck fractures is estimated to be between 600 and 900 per per year [2].

While arthroplasty is the preferred treatment in elderly patients with displaced intracapsular proximal femur fractures, in younger patients or stable fracture patterns in the elderly, joint preserving treatment with internal fixation may be favored [3,4,5,6,7,8]. However, complications that can occur after internal fixation, including excessive fracture shortening, varus collapse, avascular necrosis, and screw perforation leave room for improvement with internal fixation treatment methods [9,10,11,12,13,14]. Furthermore, variability among observed failures of the implants utilized for internal fixation of intracapsular hip fractures suggests an opportunity for optimization of implant design [9,10,11,15,16]. Both, cannulated femoral neck screws as well as the concept of sliding hip screws find many supporters. Biomechanically, the sliding hip screw concept appears to be more stable [17]. On the other hand, blood flow in the femoral head may be less impaired by cannulated femoral neck screws [18]. The large-scale FAITH trial was unable to give a qualified recommendation for one of the surgical techniques over the other [19]. In this context, the question arises whether a combination of concepts such as the Targon^®^ FN (B.Braun AG, Melsungen, Germany) could constitute a superior construct resulting in improved outcome. 

In 2007, the Targon^®^ FN system (B.Braun AG, 34209 Melsungen, Germany) was developed by Parker MJ et al., which integrated a telescoping mechanism in each of the head-neck screws (TeleScrew), aimed at allowing more controlled fracture collapse and minimizing the risk of cut-through or backing out of screws. Moreover, locking fixation to the femoral shaft provides greater rotational stability to the construct. Rotational instability and strength of femoral head fixation have shown to be associated with tendencies for femoral neck shortening, fracture collapse, and construct failure [12]. 

Several studies have shown promising early results regarding complications, non-union, and revision rates when using the Targon FN System [20,21,22,23]. In particular, the Targon FN system has shown promise in terms of lower risk of revision or re-operation compared to traditional cannulated screw fixation methods [21,24,25,26]. Other studies, however, report equivocal rates of complications with this implant comparted to the established treatment standards including cannulated screws and hemiarthroplasty [27,28]. Of interest, a high rate of elective implant removal for iliotibial band (ITB) related lateral hip pain has been described by Takigawa et al. using the Targon FN system [23].

The primary aim of this study was to evaluate the number of complications and re-operations following fixation of intracapsular proximal femoral fractures with the Targon FN system and identify risk factors for complications or re-operations. The secondary aim was to identify if lateral prominence of the implant as referenced to the lateral border of the vastus ridge was a specific risk factor for elective plate removal. 

## 2. Materials and Methods

This article was written in accordance with the STROBE-statement [29].

### 2.1. Patients 

This study is a retrospective case series of all consecutive patients older than 18 years treated for an intracapsular proximal femoral fracture with a dynamic locking plate system (Targon^®^ FN) at a single level 1 trauma center in Switzerland between 2010 and 2017. Imaging and patient data were extracted from electronic medical records. All patients received preoperative plain radiographs of the pelvis and a lateral view of the injured hip/proximal femur, intraoperative fluoroscopic images of the operative hip, as well as post-operative plain radiographs of the pelvis and hip. Patients treated for extracapsular fractures with follow up of less than three months or missing imaging and medical data were excluded. 

### 2.2. Implant, Surgical Technique, Rehabilitation and Follow Up

**Figure 1:**
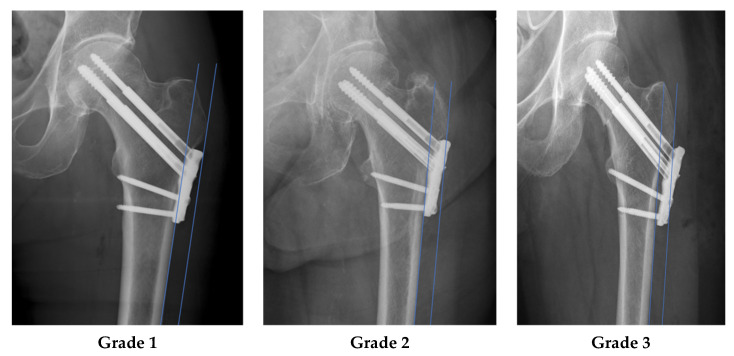
The figure shows the measurement of the plate prominence. One line is parallel to the lateral cortex of the femur, the second line is parallel to the first line and tangential to the distal portion of the trochanter major. In Grade 1 the plate is not cut by the tangential line. In Grade 2, the plate is intersected by the tangential line, in Grade 3 parts of the implant are lateral to the tangential line.

The Targon^®^ FN system (B.Braun AG, Melsungen, Germany) consists of a contoured titanium locking plate, with up to four 6.5 mm telescoping titanium sliding screws (TeleScrews) for fixation into the femoral neck, and two 4.5 mm distal locking screws for fixation to the femoral shaft. The TeleScrews have an integrated telescoping limit of 10–20 mm to prevent the risk of excessive screw back out or collapse (Figure 1).

Patients were operated under general anesthesia in the supine position. All patients received a single weight-based dose of cefazolin 30 to 60 min prior to surgery for antibiotic prophylaxis. All procedures were performed under fluoroscopic guidance. For displaced fractures, closed reduction on traction table was first attempted. Open reduction was performed at the discretion of the operating surgeon if an adequate reduction could not be achieved by closed means. For implantation of the Targon implant, a direct lateral approach to the femur was performed by either a trans-vastus approach or by elevation of the vastus lateralis along the posterior boarder of the muscle, depending on the surgeon’s preference. The specific surgical technique for the implant system was performed according to the description of Parker MJ et al. [30].

The postoperative rehabilitation protocol consisted of early active-assistive range of motion at the hip joint. Immediate, full weight bearing as tolerated was allowed for stable and non-displaced fractures (Garden I and II) in elderly patients not able to tolerate restricted weightbearing. For patients <65 and those with displaced fractures (Garden III and IV), a 6 to 12-week period of partial weight bearing was advised. All patients used crutches for a minimum of six weeks. At the discretion of the treating surgical team, patients were allowed to wean from crutches if clinical and radiological evaluation showed no secondary displacement and signs of fracture healing at follow-up.

According to our protocol, patients were evaluated postoperatively both radiographically and clinically at six weeks, three months, six months, and one year after surgery. Longer follow up was conducted as clinically necessary, however, if no clinical or radiographic complication had occurred at the 1-year mark, patients were discharged from routine surveillance.

### 2.3. Data Analysis

Demographic data were collected from of the electronic medical record and operative report including age, sex, smoking status, ASA-Score defined according to the American Society of Anaesthesiologists, dementia (yes/no), diabetes (yes/no), time to operation (from first X-ray until skin incision) (min.), type of reduction (open versus closed versus not necessary), on-call-operation (between 5 pm and 7 am), and number of TeleScrews used (two to four). A diagnosis of osteoporosis was assigned to patients with a T-score <2.5 if a Dual-energy X-ray absorptiometry (DXA) scan was available for review in the clinical record. Table 1 gives an overview of the parameters collected.

Two fellowship-trained trauma surgeons (B.L. and F.B.) evaluated and classified all pre- and postoperative radiographs. The fractures were classified using the AO and Garden fracture classification systems [31,32]. Garden type I and II fractures were categorized as non-displaced and Garden type III and IV as displaced.

The neck-shaft angle was measured on the first postoperative X-ray, one day after surgery using a digital goniometer according to the technique described by Wilson et al. [33]. A neutral neck-shaft angle was assigned to measurements between 120° and 135°, varus if less than 120°, and valgus if more than 135°. The lateral X-ray was used to detect any residual ante- or retro angulation at the fracture site after fixation. The cortical index was also measured on the first postoperative X-ray using a digital ruler, defined as the ratio of cortical width minus endosteal width divided by cortical width at a level of 100 mm below the tip of the lesser trochanter on the anteroposterior radiograph based on the description of Nash et al. [34]. The tip–apex distance was measured between the tip of the nearest TeleScrew to the apex of the femoral head in anteroposterior and axial view referring to the publication by Baumgaertner MR et al. [35]. The result was calibrated by the known lateral diameter (6.5 mm) of the TeleScrew. 

**Figure 2:**
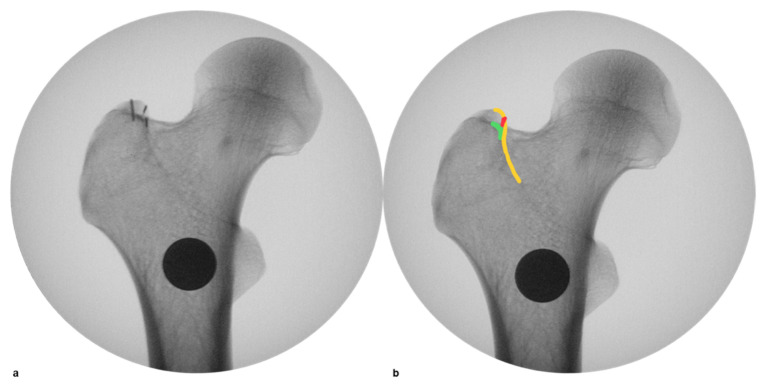
The best available anterior posterior hip image was selected based on the profile of the greater trochanter, specifically radiographic overlap of the intertrochanteric ridge and lateral wall of the piriformis fossa. (**a**): The antero- and postero-superior borders of the greater trochanter overlap in the Cortical Overlap View, this coincides with an overlapping of the easily recognizable intertrochanteric crest and density line of the piriform fossa. (**b**): Yellow marks the intertrochanteric ridge. Green marks the density line of the piriform fossa. Red marks the posterior-superior border of the greater trochanter [36].

If patients had a follow-up of more than 3 months, fracture healing, and no or only category A complication, the position of the lateral plate relative to the vastus ridge was measured and classified using the available intraoperative and postoperative radiographs (Figure 2). The best available anterior posterior hip image was selected based on the profile of the greater trochanter, specifically radiographic overlap of the intertrochanteric ridge and lateral wall of the piriformis fossa [36]. Using this image, a line was created parallel with the lateral cortical border of the femoral diaphysis and tangential to the most lateral border of the vastus ridge. Plates that were positioned medial to this line were classified as Grade 1. Any plate where the most proximal aspect of the plate intersected the line was classified as Grade 2, and plates positioned lateral to the line was classified as Grade 3 (Figure 1). 

The time at which full weight bearing was permitted, as documented by the surgeon in the medical record, was categorized parametrically to either immediately, 6 weeks, 12 weeks, or greater than 12 weeks.

All surgical complications mentioned in the operative report (for example, damage to vascular, nerves, or other structures, additional implants, conversion to arthroplasty) were categorized in this study as intraoperative complications.

Postoperative complications were collected from the medical record and the corresponding x-rays at the post operative follow-up. Only complications directly related to the surgical site were included. 

We defined persistent mechanical irritation of the iliotibial band as category A complication. Other complications including plate or screw breakage, screw perforation into the hip joint, secondary loss of reduction (varus deformity with neck-shaft angle <120° or valgus deformity with neck-shaft angle >135°), development of a pathological femoral offset leading to symptomatic femoroactetabular impingement (FAI) and superficial or deep surgical site infections (SSI) were defined as category B. Superficial surgical site infection was defined as an infection of the surgical site involving skin and subcutaneous tissue, occurring within 30 days after surgery. Deep SSI involved soft tissues deep to the subcutaneous tissue and could occur up to one year after surgery [37]. A diagnosis of avascular necrosis was defined by Steinberg stage two or greater on any of the follow up radiographs [38]. Implant failure was defined by any damage of the implants noted on radiographs including breakage of the plate or breakage or loosening of any screws. Non-union was defined if the fracture showed no evidence of bony fusion of at least 2 cortices on conventional X-rays in two planes after 6 months.

Any re-operation at the same surgical site performed after the index operation, including implant removal, was recorded. 

### 2.4. Statistical Analysis

All computations were done with the Statistical Package for Social Sciences (SPSS), version 22 (IBM SPSS Statistics, USA). Continuous data were presented as means with corresponding standard deviation (SD) when normally distributed. In other cases, median with interquartile range (IQR) was used. Categorical variables were presented as counts and corresponding percentages. Differences in continuous variables were analyzed using the independent T-test for normally distributed and Mann–Whitney U test for non-normally distributed variables. Differences in categorical variables were analyzed using the Fisher’s exact or Chi-Square test, respectively. 

Univariate risk factor analysis was performed using logistic regression and presented as odds ratio’s (OR) with corresponding 95% confidence interval (95% CI). *p <* 0.05 was considered statistically significant. No multivariate analysis was possible due to low number of events in the regression model.

## 3. Results

The study included a total of 83 cases of proximal femur fractures treated with the Targon^®^ FN system between 2010 and 2017. Of these, one was excluded due to a pertrochanteric fracture pattern and ten were excluded due to a follow-up of less than the minimum of three months. Mean follow-up was 19.7 months with a range from 3 to 92 months. A subgroup of 20 patients has a follow-up of less than 12 months. The baseline demographics and risk factors are listed in Table 1.

### 3.1. Complications and Re-Operations

Forty of seventy-two cases (55.6%) recovered without any category A or B complications and required no unplanned re-operation during the complete follow-up period. 

In the 32 remaining cases, at least one complication occurred. The details of these complications can be found in Table 2. Thirty-two cases required one re-operation, and nine patients required more than one re-operation. A total of 46 re-operations were performed. The summary of the re-operations is listed in Table 3.

### 3.2. Analysis of Risk Factors for Complications

Risk factors for complication (except irritation of the iliotibial band) including ASA-Score, energy of trauma, time to postoperative weight bearing, postoperative caput-collum-diaphyseal (CCD) angle did not show significant differences comparing patients who experienced a complication to patients without complications in univariate analysis. Only age 60 years and younger was observed to be an independent risk for mechanical irritation of the iliotibial band in a univariate logistic regression analysis (OR 8.8, 95% CI 2.3–34.5, *p* = 0.001).

### 3.3. Analysis of Risk Factors for Re-Operation

Statistical analysis showed that age 60 years and older was significantly related to a lower chance of a second operation (OR 0.25, 95% CI 0.098–0.637, *p* = 0.004). In cases with displaced femoral neck fractures (Garden Type III, IV), there was a significantly higher risk of re-operation (OR = 2.73, 95% CI 1.09–6.83, *p* = 0.03). Moreover, patients that did not reach full weight bearing until after 12 weeks had a significantly higher re-operation rate (OR 3.44, 95% CI 1.07–11.07, *p* = 0.04). Finally, there was a significant correlation between a valgus (>135°) postoperative CCD angle and rate of re-operations (OR 3.11, 95% CI 1.20–8.10, *p* = 0.02). Gender, smoking status, diabetes, osteoporosis, trauma energy did not show a significant association to higher re-operation rates. A complete list of re-operation risk factors is shown in Table 1.

### 3.4. Plate Position as a Risk Factor for Plate Removal

Fifty-three cases with a follow-up of more than 3 months, fracture union, and no or only category B related complications were analysed to assess the plate position relative to the lateral vastus ridge line described. The plate was classified as Grade 1 in 11 (20.8%) cases, Grade 2 in 36 (69.8%) cases, and Grade 3 in 6 (11.3%) cases. Of the 17 patients who underwent elective plate removal due to lateral hip pain and ITB irritation, 1/11 (9%) was classifed as Grade 1, 14/36 (38%) Grade 2, and 2/6 (33%) grade 3. Relative to Grade 1, the odds ratio for plate removal for Grade 2 was found to be 6.36 (95% CI 0.73–55.30, *p* = 0.064) and 5.00 (95% CI 0.35–71.90, *p* = 0.21) for Grade 3. Although not statistically significant, the data demonstrated a higher risk of plate removal with higher grade of plate position. In all patients, pain resolved after removal of the implant.

## 4. Discussion

### 4.1. Key Results

In summary, thirty-four of seventy-two cases (47.2%) treated for an intracapsular femoral neck fracture using the Targon FN system had at least one complication. The most common complication was mechanical irritation of the ITB (23.6%) followed by screw perforation (6.9%), avascular necrosis of the femoral head (5.6%) and non-union (5.6%). Statistical analysis identified patient age 60 years and younger to be an independent risk for mechanical irritation of the iliotibial band. According to statistical analysis, displaced femoral neck fractures (Garden Type III, IV), delayed full weight bearing after 12 months, and a higher postoperative CCD angle (>135°) were significantly associated with re-operations. Although not statistically significant, there is a higher odds ratio for removal of implants in a more laterally prominent position. 

### 4.2. Limitations

The present study has limitations. There was a relatively small number of cases at a single center, with a retrospective study design, and no control or comparison group to other fixation methods. A subgroup of 20 patients has a follow-up of less than 12 months. In this collective, we must assume that we even have underestimated the rate of avascular necrosis, delayed bone healing and irritation of the ITB. Additionally, if patients experienced complication but cared for at outside institutions, the present study would not have captured those events.

Regarding radiographic assessment, there was no standard to ensure the consistency of the AP view acquired used to classify the position of the plate. This was best mitigated by using the best available image of all postoperative and intraoperative views. Furthermore, the technique to determine the TAD was developed for the sliding hip screw with a single screw placed in the femoral head and neck. It is unclear if the concept of TAD applies to an implant consisting of 3 to 4 sliding screws. In the current study the CCD angle was measured on plain ap radiographs. In some cases, it was difficult to define the center of the femoral head for example due to head deformities in cases of osteoarthritis of the hip. Moreover, the definition of the center of the femoral neck was occasionally complicated by posttraumatic deformities. Due to the potential of radiographic technique to confound measurement the clinical significance behind the correlation of high CCD >135° and higher rates of re-operation cannot be made.

### 4.3. Interpretation

Traditional fixation constructs for intracapsular femoral neck fractures include multiple cannulated screws or sliding hip screw and plate designs. Each of these constructs present unique modes and rates of failure. The literature reports complication rates between 10 and 33% and re-operation rates between 30 and 50% for the treatment of intracapsular proximal femoral fractures using these traditional methods [9,10,11,19,21,30,39,40,41]. The Targon FN system was therefore designed to blend the best of both the cannulated screw and sliding hip screw designs by providing multiple smaller diameter screws for fixation into the femoral neck combined with the rigidity of a fixed angled side plate, while allowing for controlled fracture collapse [24].

Literature comparing the Targon FN system to traditional fixation constructs in terms of complication rates is inconsistent. A recent study of two-thousand femoral neck fractures comparing Targon FN to cannulated screws, Alshameeri et al. reported screw cut out in 0.6%, AVN in 7.0% and non-union in 9.5% of all cases treated with the Targon FN system. In cases with displaced femoral neck fractures screw cut out was found in 0.9%, AVN in 8.9% and non-union in 14.4 %. They found the Targon FN System to be associated with lower rates of non-union compared to the cohort treated with cannulated screws. Similar results in smaller cohorts of patients had been previously published by this group. Of note, the authors for these studies included individuals responsible for the design of the Targon FN system implant. At an institution independent from the design institute, Osarumwense et al. reported results in favor of the Targon FN [24]. In this study, a 9% complication rate during a 24 month follow up period. Nearly the same results were published in a study by Sass et al. where they found a 9% rate of complications treated with the Targon FN [42]. In contrast, Griffin et al. found no clinical difference in the risk of revision surgery between the Targon FN (n = 51) and cannulated screw fixation (n = 123) for treatment of intracapsular hip fractures [28]. In this study, 31% of the Targon FN patients and 36% of patients in the cannulated screw group underwent re-operation within 12 months postoperatively.

While in the present study the rate of non-implant associated complication including AVN and non-union is within a similar range as previously reported with this implant, a high rate of implant associated complications due to mechanical irritation of the ITB and lateral hip pain was observed. To our knowledge, there is only one study discussing this finding. Takigawa et al. reported a 10.9% rate of elective implant removal after non-displaced and 48.3% after displaced fractures, for what was reported as discomfort around the implant. In the discussion the authors hypothesized that the size of the Targon FN plate may be too large for the Asian population and could lead to increased irritation of the soft tissue around the plate [23]. The high rate of mechanical irritation in the present study shows that the problem seems not to be limited to an Asian population and potentially due to the position of the plate. Although this study was underpowered to detect a significant correlation between higher grades of implant prominence and rates of implant removal, there was a higher odds ratio of 5 to 6 times for more prominent implant position. Moreover, statistical analysis identified patient age of 60 years and younger to be an independent risk for mechanical irritation of the ITB. We acknowledge that with the use of locking screws in the distal aspect of the plate, there is the possibility of leaving the plate prominent if the surgeon is not conscious to ensure that the plate lies flush against the lateral cortex when applying these locking screws. There could, therefore, be a learning curve associated with this implant resulting in plate prominence that was not accounted for in the current study, but potentially a subject of future study.

The present study aimed to identify risk factors influencing the rate of re-operation. Younger age, fracture dislocation, time to postoperative weight bearing of more than twelve weeks and postoperative caput–collum–diaphyseal (CCD) angle were identified as factors increasing the rate of re-operation. Age less than 60 was the only factor influencing the rate of implant removal. In most other studies the opposite is reported. Carpinetero et al. for example, reported that patients older than 65 years have pre-existing medical comorbidities and thus a higher risk of re-operations and complications (non-unions and avascular necrosis). In our opinion, the higher re-operation rate for younger patients is attributed to a potentially higher activity level of younger patients and resulting in more mechanical irritation around the implant, and possibly more symptomatic than in older, less active patients. We purport that this could lead to a bias toward implant removal and is a reason for younger age as a risk factor for re-operation for implant removal observed in this study. 

This study also found a significant correlation between fracture displacement and the likelihood of re-operations. This finding is in line with L.T. Nilsson et al. who reported on a prospective series of femoral neck fractures of 138 patients finding fracture displacement as the only predictive factor of complications [5]. Comparable findings were made by A.Alho et al. in a study with 149 cases of femoral neck fractures. They identified displaced femoral neck fractures to be a risk factor for impaired fracture healing and advocated arthroplasty instead [6]. Parker et al. compared 56 patients with displaced intracapsular fractures randomly treated with hemiarthroplasty or the Targon FN. He found significantly higher reoperation rates and higher postoperative pain in the internal fixation group [43]. 

Delay to weightbearing beyond 12 weeks was also found as a risk fractor for re-operation. In fact, recent studies recommend an early transition to full weight bearing [8,44]. A study by Kolaczko et al. showed that limiting weight bearing for more than 8 weeks may negatively affect bone healing [44]. In turn, this could lead to prolonged higher loads on the implant and thus promote implant failure. However, bias toward cautious aftercare may be influenced by many factors including the experience of the treating surgeon, patient age, general medical condition of the patient, a somewhat less than perfect reduction or implant position. Therefore, this finding may be a surrogate parameter for other risk factors rather than a risk factor on its own.

## 5. Conclusions

In summary, there is a high complication rate for patients treated for intracapsular femoral neck fracture using the Targon FN system. The most common complication was mechanical irritation of the iliotibial band around the implant. Age 60 years and younger was an independent risk factor for ITB irritation requiring plate removal. We hypothesize this is associated with lateral plate prominence and higher activity levels in younger patients. Statistical analysis showed that patient age, fracture dislocation, time to postoperative full weight bearing were risk factors for re-operation. In our opinion, if the Targon FN system is utilized, plate position should be as flush as possible to avoid mechanical irritation of the ITB.

## Figures and Tables

**Table 1:** Baseline Characteristics and Risk Factors (n = 72).

Patient Dependent Factors		Hospital Dependent Factors	
Sex, No. (%)		Time to surgery hours (SD)	
Male	35 (48.6)	Mean	19.99 (20.65)
Female	37 (51.4)	postoperative CCD angle No.	
Age, y		<125	5 (6.94)
Mean (SD)	61.36 (16.35)	125–135	29 (40.28)
Median (range)	60.50 (25–89)	>135	38 (52.78)
Age group, No. (%)		measured TAD (SD)	
<65 y	42 (58.33)	Mean	18.79 (5.05)
≥65 y	30 (41.66)	Reduction No. (%)	
ASA, No. (%)		Open	23 (31.94)
I	10 (13.9)	Closed	29 (40.28)
II	38 (52.8)	Not necessary	20 (27.78)
III	23 (31.9)	Time to full weight bearing weeks No. (%)	
IV	1 (1.4)	Immediately	27 (37.50)
V	0 (0)	6 weeks postoperative	26 (36.11)
Diabetes, No. (%)		10–12 weeks postoperative	19 (26.39)
Type-I	1 (1.4)	Out of office operation No. (%)	
Type-II	3 (4.2)	Yes	33 (45.83)
No	68 (94.4)	No	39 (54.17)
Dementia No. (%)			
Yes	5 (6.9)		
No	67 (93.1)		
Osteoporosis No. (%)			
Yes	10 (86.1)		
No	62 (13.9)		
Smoking status, No. (%)			
Yes	17 (23.6)		
No	55 (76.4)		
AO fracture classification No. (%)			
31-B1	30 (41.7)		
31-B2	35 (48.6)		
31-B3	7 (9.7)		
Garden fracture classification (%)			
Non-displaced	41 (56.9)		
Displaced	31 (43.1)		
Trauma intensity * No. (%)			
Low	51 (70.8)		
High	21 (29.2)		

* Trauma intensity was classified according to ATLS as “low” for fall heights up to 2 meters or speed deltas up to 30 km/h, and as “high” above these values.

**Table 2:** Detailed Listing of Complications with Indication of Underlying Cause. * Percentage of the underlying complication referred to 72 cases. ** Reoperations are indicated by the event that led most likely to the intervention because an associated complication may also lead to a reoperation.

Complications	Cat. A	Cat. B	All	% *	Reoperations **
Hematoma and bleeding					
hematoma	-	1	1	1.4	3
Soft tissue					
tractus irritation	17	-	17	23.6	17
Reduction					
Secondary loss of reduction	-	2	2	2.8	2
Plate and screws					
screw perforation through the cortex of the femoral head	-	5	5	6.9	5
Loosening Tele Screw	-	2	2	2.8	5
Loosening Screw base plate	-	1	1	1.4	1
Osseus disorders					
avascular necrosis of the femoral head	-	4	4	5.6	5
nonunion	-	4	4	5.6	4
postoperative femoroactetabular impingement	-	2	2	2.8	4
Total number	17	21	38	52.8	46

**Table 3:** The table gives a summary of the resulting 46 re-operations. In the left column, all procedures we performed are listed. If one procedure was performed in one operation the number of cases is found in a diagonal manner (light green fields). Those cases where two procedures were performed in one operation are shown in the lower left of the table (light blue fields). ITB = Ileotibial band.

	Complete Implant Removal	Partial Implant Removal	Total Hip Arthroplasty	Hip Arthroscopy	Revision 90° Blade Plate	Girdlestone Procedure	Revision Total Hip Replacement	Wound Revision	Revision of ITB
**Complete implant removal**	**20**								
**Partial implant removal**		**4**							
**Total hip arthroplasty**	**6**		**2**						
**Monopolar hip arthroplasty**	**1**								
**Hip arthroscopy**	**1**			**1**					
**Cement spacer interposition**	**1**								
**Removal of cement spacer**			**1**						
**Valgus osteotomy with 90° blade plate**	**1**								
**Revision 90° blade plate**					**1**				
**Girdlestone procedure**						**1**			
**Exchange of one TeleScrew**		**1**							
**Revision total hip replacement**							**1**		
**Wound revision**								**3**	
**Revision of ITB**									**1**

## Data Availability

Data supporting reported results are stored by the corresponding author.
